# Zika-Related Microcephaly and Its Repercussions for the Urinary Tract: Clinical, Urodynamic, Scintigraphic and Radiological Aspects

**DOI:** 10.3390/v14071512

**Published:** 2022-07-11

**Authors:** Rômulo Augusto Lucena de Vasconcelos, Ricardo Arraes de Alencar Ximenes, Adriano Almeida Calado, Celina Maria Turchi Martelli, Andreia Veras Gonçalves, Elizabeth Bailey Brickley, Thalia Velho Barreto de Araújo, Maria Angela Wanderley Rocha, Demócrito de Barros Miranda-Filho

**Affiliations:** 1 Pós-Graduação em Ciências da Saúde, Universidade de Pernambuco, Rua Arnóbio Marques, 310, Santo Amaro, Recife 50100-130, Brazil; raaximenes@uol.com.br (R.A.d.A.X.); caladourologia@yahoo.com.br (A.A.C.); andreiaverasg@gmail.com (A.V.G.); mangelarocha@uol.com.br (M.A.W.R.); 2 Departamento de Medicina Social, Universidade Federal de Pernambuco, Recife 50670-901, Brazil; thalia.velhobarreto@gmail.com; 3 Instituto de Pesquisa Aggeu Magalhães, Fundação Oswaldo Cruz (Fiocruz), Recife 50740-465, Brazil; turchicm@gmail.com; 4 Department of Infectious Disease Epidemiology, London School of Hygiene and Tropical Medicine, London WC1E 7HT, UK; elizabeth.brickley@lshtm.ac.uk

**Keywords:** congenital Zika syndrome, microcephaly, Zika virus, urodynamics, urinary tract diseases

## Abstract

Aims: Describing the urodynamic parameters of children aged 3 to 5 years with microcephaly related to congenital Zika syndrome and verifying the association with clinical, imaging and neurological characteristics. Methods: From October 2018 to March 2020, children with Zika-related microcephaly underwent urological, ultrasonographic and urodynamic evaluation. In selected cases, complementary exams such as urethrocystography and scintigraphy were performed. The children also underwent a complete neurological evaluation. To compare frequency between groups, we used Pearson’s chi-squared test or Fisher’s exact test. Results: This study evaluated 40 children, of whom 85% were 4 years old, and all had abnormalities on the urodynamic study, with low bladder capacity (92.5%) and detrusor overactivity (77.5%) as the most frequent findings. Only three children had ultrasound abnormalities, but no child had cystographic or scintigraphic abnormalities, and the postvoid residual volume was normal in 80% of cases. In spite of a frequency of 67.5% of intestinal constipation, there was no record of febrile urinary tract infection after the first year of life. All children presented severe microcephaly and at least one neurological abnormality in addition to microcephaly. The homogeneity of the children in relation to microcephaly severity and neurological abnormalities limited the study of the association with the urodynamic parameters. Conclusions: Urodynamic abnormalities in children aged 3 to 5 years with Zika-related microcephaly do not seem to characterize a neurogenic bladder with immediate risks for the upper urinary tract. The satisfactory bladder emptying suggests that the voiding pattern is reflex.

## 1. Introduction

In 2015, the Brazilian Ministry of Health identified a notable increase in cases of microcephaly, initially in the northeastern state of Pernambuco (PE), and a Public Health Emergency of National Importance was declared [1]. With the aim of conducting research to support the public health response, the Microcephaly Epidemic Research Group (MERG) was created in 2016 in Pernambuco, bringing together researchers from the Aggeu Magalhães Research Center of the Oswaldo Cruz Foundation—PE, the Federal University of Pernambuco, the University of Pernambuco (UPE) and the London School of Hygiene & Tropical Medicine. Early research by MERG confirmed the association between microcephaly and Zika virus (ZIKV) infections during pregnancy [2], corroborated by subsequent studies [3,4,5], and provided an initial description of the broader congenital Zika syndrome (CZS) [6].

Even with the detection of cases of ZIKV infection on all continents, either by autochthonous transmission or importation through travelers, Brazil remains the country with the highest number of cases. According to the last epidemiological bulletin of 2022, 11,043 cases were reported in Brazil, with a cumulative incidence (CI) of 5.16, followed by Guatemala with 1068 cases (CI = 5.85) and Paraguay with 315 cases (CI = 4.36) [7].

Although the hallmark characteristics of CZS include the impairment of brain development usually associated with microcephaly, additional clinical and radiological findings, such as craniofacial disproportion, spasticity, dizziness, irritability, brainstem dysfunction, dysphagia, neurodevelopmental delay and sequelae such as epilepsy may be present [8]. Manifestations involving other systems (e.g., cryptorchidism and endocrine dysfunction) have also been described [9,10]. Among adults, Guillain–Barré Syndrome [11,12], myelitis and peripheral neuropathy can also be associated with ZIKV infections [13].

The function of the LUT is regulated and guided by the nervous system at the cerebral cortical, brainstem, spinal cord and peripheral nerve levels. The LUT matures by 5 years of age, when the adult pattern of voiding control is typically established. Until this age, voiding and urodynamic parameters usually seen in children are not considered pathological (i.e.,: urinary incontinence and detrusor vesico-sphincteric dyssynergia), and additional exams may be necessary to evaluate the urinary tract and better characterize the occurrence of NB. These children urinate in a reflex pattern as neurovesical and urinary tract function still matures [14].

Different patterns of NB can be expected in patients with neurological damage, depending on the affected sites and severity. NB can lead to renal impairment and incur socioeconomic costs related to urinary incontinence [14]. Children with NB generally present significant and varied neurological impairment, requiring the characterization of the functioning of the LUT and its impact on the kidneys [15,16].

In 2018, Monteiro et al. described findings suggesting functional impairment of the lower urinary tract (LUT) in 22 children with Zika-related microcephaly (ZRM) evaluated by a urodynamic study (UDS). All children had urodynamic abnormalities, with detrusor overactivity (DO) with vesico-sphincteric synergism as the most frequent finding (95.5%) [17]. These findings were maintained in a subsequent study of the same group [18]. These findings were characterized as Neurogenic Bladder (NB) with risk for the upper urinary tract. In a recent systematic review, the first studies addressing to LUT function presented discordant findings and methodological criticisms [19].

This study aims to describe urodynamic parameters of children aged 3 to 5 years with ZRM and to verify the association with clinical findings and imaging and neurological characteristics in order to contribute to a better understanding of this condition in this population.

## 2. Materials and Methods

This is a cross-sectional study, including children followed up as part of the MERG Pediatric Cohort (MERG-PC), composed of children aged 3 to 5 years with ZRM followed up at the Oswaldo Cruz University Hospital—UPE from October 2018 to March 2020. The study included children with ZRM referred for urological evaluation according to the cohort assessment protocol, in spite of urinary complaints or not. Children were considered with ZRM if they were born during the microcephaly epidemic (from May 2015 to April 2017) with microcephaly diagnosed either at birth or during the first months of life; presenting with either laboratory evidence of ZIKV infection or imaging abnormalities compatible with CZS [20]. The exclusion criteria were acquired neurological lesions, malformations of the genitourinary tract that could prevent examinations and acute urinary tract infection (UTI).

A total of 61 children underwent urological evaluation during the study, and 40 underwent urodynamic and ultrasonographic evaluation.

Urine cultures were not collected prior to the UDS, but all participants received antibiotic prophylaxis with 1st generation cephalosporin. The UDS (Uranus II^®^/Alacer^®^) was conducted by the same pediatric urologist through the insertion of 2 catheters via the urethra for infusion of saline solution at 1 mL/kg/min at room temperature (6Fr) and measurement of bladder pressure (4Fr). During the voiding phase, due to reflex voiding, the catheters were maintained in their position to avoid interference with the urinary stream. Abdominal pressure was measured by a rectal balloon with 10 mL of saline solution. The children were in supine position at the level of the transducers. Electromyography for sphincteric activity was not performed due to equipment limitations.

The urodynamic parameters evaluated were: bladder capacity (BC), which was considered low when <65% of the estimated BC registered at the time of loss of detrusor compliance or triggering of detrusor overactivity; detrusor compliance (DC), which was calculated by the ratio of final volume/final detrusor pressure in the absence of detrusor overactivity and considered low when <20 mL/cmH_2_O; detrusor overactivity (DO), which was considered present when the transient increase in detrusor pressure was >15 cmH_2_O, according to the definition adopted by Monteiro et al [17]; detrusor leak-point pressure (DLPP), which was considered high when ≥40 cmH_2_O and was registered at the start of the urinary stream, considering that children under 5 years of age void in a reflex pattern; and postvoid residual volume (PVRV), which was considered high when there was >10% of the EBC and registered by active aspiration of the bladder before removing the catheters used for the exam. An international standardization was adopted, and results were revised by another pediatric urologist who did not participate in the exams [21].

The sonographic characteristics evaluated were: hydronephrosis; ureteral dilation (distal ureter diameter ≠0 cm); bladder wall thickness (thick, if ≥0.3 cm with full bladder); and bladder diverticulum.

The characteristics of the cystourethrography investigated were: bladder neck (open or closed); vesical aspect (abnormal, if piriform or Christmas tree); bladder diverticulum; and vesicoureteral reflux.

The characteristics of the scintigraphic evaluation with dimercaptosuccinic acid (DMSA) were: renal function (decreased, if uptake <45%) and kidney scars.

Information on previous febrile UTI and intestinal constipation was obtained through direct questioning to mothers or caregivers. Intestinal constipation was considered present when the child was under medical treatment. Information on neurological examination was obtained from MERG neurological form.

Microcephaly degree was classified as mild, when the z-score for cephalic perimeter < 2 SD, or severe, when the z-score for cephalic perimeter < 3 SD [22].

We retrieved the results of the electroneuromyography of 5 children who had performed this exam due to indication of neuropediatrician to evaluate peripheral nerve function and 15 electroencephalograms (EEG) from MERG neurological form.

For the description, the frequency for categorical variables and mean and standard deviation for continuous variables were presented. For frequency comparisons, Pearson’s chi-squared tests, or Fisher’s exact tests were used, considering the difference to be statistically significant when *p* < 0.05. We used SPSS for Windows^®^, version 18.0—Statistical Package for the Social Science^®^.

All parents/guardians signed an informed consent form. This study was approved by the Oswaldo Cruz Hospital Ethical Committee (CAAE: 94544518.2.0000.5192).

## 3. Results

From the 40 children who underwent urodynamic and ultrasonographic evaluation, 11 participants had voiding cystourethrography and DMSA scintigraphy indicated, as they presented findings that could compromise the upper urinary tract (UUP) (i.e., low DC, high DLPP, high PVRV and/or abnormal ultrasonography), being referred for additional treatment, too. Only five participants returned these exams. The age of study participants ranged from 3 to 5 years old, 85% being 4 years old. From 40 participants, 23 (57.5%) were male, and 36 (92.3%) were from the metropolitan region of Recife. All participants presented severe microcephaly, with z-scores ranging from −3.27 to −12.42.

Table 1 shows the results of specific urological exams, relevant medical history and neurological characteristics. All children showed abnormal UDS results, and three children had abnormal ultrasound results (presence of vesical diverticulum in two cases and thickening of the bladder wall in one case). Five children performed voiding cystourethrography and five performed DMSA renal scintigraphy, with no abnormal finding in any of the exams. No child had UTI prior to the UDS, but it was previously documented in 10 children, all in the first year of life. Intestinal constipation was identified in 27 (67.5%) participants who underwent the clinical evaluation.

Low BC and DO were the most frequent urodynamic findings (92.5 and 77.5%). The first is usually associated with the second. The most frequent pattern in DO was high detrusor pressure with low DLPP. PVRV was increased in eight children (Table 1). Table 2 presents a summary of the urodynamic findings for all children.

Low BC was the only urodynamic parameter associated with the presence of a history of febrile UTI (*p* = 0.012). No urodynamic parameters were associated with the presence of constipation (Table 3 and Table 4).

From 27 children with intestinal constipation, 8 (20%) had a history of febrile UTI. There was no association between the presence of constipation and a history of febrile UTI (*p* = 0.451).

## 4. Discussion

This study presents the results of the urodynamic and ultrasonographic evaluation of 40 children aged 3 to 5 years old with ZRM followed up by the MERG and, for the first time, compares these findings with the neurological characteristics of these children. Previous studies included either children aged fewer than 41 months [17,18,23] or aged from 35 to 47 months, but with a smaller sample size [24]. As age is an important factor for LUT function development [14], this first report with children older than 2 years old exclusively offers new information about NB in children with ZRM.

A frequency of 100% for abnormalities in the UDS of these children is compatible with reports in the literature, which, although scarce, range from 92.6 to 100%. DO with high pressure and low BC found for age were the most frequent patterns, which is also consistent with previous reports [17,18,23,24]. The high frequency of DO found in our study (77.5%) influenced BC by triggering contractions with low volumes for age. Although there are no standardized values for DC for children, this finding was present in 25% of the UDS by the established criteria, higher than the most current findings in children older than 2 years with ZRM (11.1%) [24]. Only one child did not present urination at the end of the UDS, being referred for immediate clean intermittent catheterization. Despite the frequency of overactivity with high detrusor pressure, bladder emptying was efficient (normal PVRV) and occurred at pressures below 40 cmH_2_O in the majority of cases, compatible with the most current findings in children older than 2 years (18.5%) [24].

Additionally, no urethrocystographic or scintigraphic abnormalities were observed, and only three patients had abnormal ultrasonography, but two of these findings were characterized as congenital diverticula due to the uniqueness and to the absence of other abnormalities. Only one patient had signs of bladder thickening. A delay in sphincter relaxation, compatible with reflex urination, could justify this urodynamic pattern without repercussions on the UUT and normal PVRV.

Medeiros et al., recently reported no hydronephrosis on ultrasonography or cases of UTI during the urological evaluation of children with ZRM [24], while a previous study in children younger than 2 years old identified UTI in 22.73% of patients and hydronephrosis in 9.09% [17]. A recent study including children from 2 to 3 years old found no hydronephrosis on ultrasonography [23]. In Southeast Asia, where ZIKV infection is not uncommon, there were no reports of functional impairment of the LUT [25]. A study comparing children with ZRM with urodynamic abnormalities who were undergoing clinical treatment did not observe any statistical difference between adherents and nonadherents to therapy in relation to the urodynamic and clinical findings studied [26]. This disparity could be explained by a modification of the neurovesical and sphincter components over time in both groups. More severe cases would be more frequent in children under 2 years of age, while the urodynamic pattern would present a lower risk to the kidneys after that age due to the acquisition of a reflex voiding pattern. The urodynamic assessment of these children can be extremely challenging, and it is possible that the nonuse of a rectal balloon during the examination in previous studies with findings of higher risk for the UUT has interfered with the records of detrusor pressures, with repercussions on the presence of DO, maximum pressure of contraction of the bladder muscles and, consequently, reduction in DC. In addition, it is possible that peculiarities of the neuromotor development of these children influence the different findings from 2 years of age onwards. The neuromotor development of children with ZRM is severely compromised; it may have been delayed from 13 months to 18 months of age [27]. In addition, other studies showed that 65% to 99.1% of the children with ZRM were at risk of neurodevelopmental delay depending on the severity of the microcephaly [28]. Our findings indicate a voiding pattern of lower risk for the UUT, as we are evaluating children between 3 and 5 years old. This assumption is also based on the absence of UTI cases during our urological evaluation and on the fact that reports of febrile UTI were only observed in 25%, with no occurrences after 1 year of life. CZS is complex, with multisystem manifestations still under investigation. The possibility of additional involvement of other regions of the nervous system could also justify distinct urodynamic and radiological findings, such as in myelitis and meningoencephalitis [13]. The spinal cord and neuronal study in this disease still lacks further evidence. The low intersection between the presence of urodynamic and ultrasonographic changes suggests a urodynamic pattern of immaturity or neurological delay, where DO would work as reflex voiding without repercussions on the UUT or on the incidence of UTI, being associated with efficient bladder emptying as recently suggested [24].

For ethical reasons, it was not possible to assemble a control group for assessing urodynamic parameters, but due to the similarity of the cortical lesion, both congenital microcephaly from other causes and cerebral palsy can be compared with ZRM [27]. The literature is scarce in both cases. In 1997, two cases with congenital microcephaly, aged three and six years, were diagnosed with high-pressure DO and vesico-sphincter dyssynergia and hydronephrosis. Both were effectively treated clinically after three months [29]. In a systematic review evaluating the urodynamic parameters of adult and pediatric patients with cerebral palsy, abnormalities were found in 84.5% of the cases, with DO (determining a low BC) present in 59% of the exams [30]. In children aged 1 to 17 years, low BC was found in 54% of the cases, DO in 35.1%, high PVRV in 13.5% and low DC in 10.8%. These children were referred for urodynamic evaluation because of the diagnosis of UTI. Still, no complications were identified in the UUT [31]. These findings, considering the recruitment of already symptomatic children and including children under 2 years of age, are close to ours and may corroborate the apparent lower risk for the UUT in these similar populations.

Low BC was the only urodynamic parameter associated with a history of febrile UTI (*p* = 0.012). This parameter is interfered with by other events, notably DO, but also loss of DC, but neither of these two parameters was associated with a history of UTI in this study. It is possible that a modification, albeit late, in the neuromotor development of these children, associated with or influencing changes in the neurovesical and sphincter components, is responsible for these findings. In younger children, distinct findings have already been reported [17]. In children with cerebral palsy, the presence of urinary retention and febrile UTI were related to the deterioration of the UUT [32]. The low frequency of these findings in our study can explain the preservation of the UUT and reinforce the possible character of development of neurological components but also of the urinary tract and a maturation of the relationship between them.

The high frequency of constipation (67.5%) in our study is consistent with the findings of another study that investigated vesicointestinal dysfunction in children aged 1 to 5 years with ZRM (80%) [33]. Constipation is a frequent finding in children with neurological abnormalities and compromised functioning of the abdominopelvic muscles [16,30,31]. Some degree of injury to motor neurons or spinal cord could justify the high frequency of this abnormality [13], as well as nutritional factors (e.g., low fiber intake) and low water intake, already described in children with cerebral palsy [34]. Despite the high frequency of constipation in our study, there was no association of this finding with any urodynamic parameter or with a history of febrile UTI. Despite the already recognized association between constipation and UTI [14], the absence of this finding strengthens the idea of a low-risk bladder for the UUT in children over 2 years of age. Azevedo de Almeida et al., studying children with ZRM, observed a frequency of UTI of 47.5% in patients with vesicointestinal dysfunction. That study found DO in 90% of patients, low DC in 35% and low capacity in 90% but PVRV ≤ 20% of cystometric capacity in 60% [33]. The urodynamic findings are close to those found in our study, despite that the ages of participants in their study ranged from 1 to 5 years old. The mechanism responsible for the low frequency of UTIs in our study may be related to adequate bladder emptying, as demonstrated by the high frequency of PVRV <10% of the EBC, but the use of a strictly clinical evaluation, without uroculture, before performing the UT may not have taken into account asymptomatic bacteriuria and/or contamination, too.

Despite the high frequency of neurological abnormalities, only increased appendicular tone and high pressure in DO (*p* = 0.038), high PVRV and decreased axial tone (*p* = 0.003) and high PVRV and presence of seizures (*p* = 0.020) showed an association. The lack of other associations may be related to the small size and, especially, the homogeneity of the sample: all participants have microcephaly with severe brain impairment, associated with a high frequency of urodynamic abnormalities and neurological findings.

As all participants presented severe microcephaly, it was not possible to study the association between urodynamic disorders and the severity of CZS.

The complexity of neurological damage in these children is already well-established, although it is still in investigation [28]. From brain abnormalities to spinal cord and neuronal injuries, the finding of severe impairment in neurological development can be reflected in a myriad of associated situations, including the dynamics of the LUT. Peripheral neuronal pathways may also be severely compromised [35,36]. Hypertonicity, observed in children with ZRM, reflects sustained activity of the motor neuron, with repercussions on joint systems, but which could explain the intensity of bladder contractions triggered by the pudendal nerve. Injuries to the afferent pathways, pontines or autonomic control systems and voiding reflex can also interfere with the fine control of the LUT activity. Especially, already recognized pontine encephalic lesions^5^ may determine reflex urination without cortical control, which may also be compromised. In the children with high PVRV, contractile deficit and/or dyssynergia mechanisms could be related to such findings. A limitation of this study was the fact that electromyography was not performed, not allowing us to infer on the existence or not of this urodynamic finding. However, the general pattern of apparently lower risk for the UUT observed in this case series, especially without the observation of renal abnormalities, low frequency of high PVRV and the presence of current UTI suggest a low frequency of dyssynergia.

The COVID-19 pandemic negatively impacted the return of these children for additional evaluation, such as DMSA and voiding urethrocystography, due to public health restrictions, but continued contact through phone calls provided no evidence of complications related to the urinary tract.

The apparent benign nature of the LUT functioning in the studied children could contribute to a less invasive initial investigation (e.g., uroflowmetry with electromyography and ultrasonography) or focused investigations on children with ZRM and other medical indications (e.g., occurrence of febrile UTI).

## 5. Conclusions

The presence of abnormalities in the UT of children aged 3 to 5 years with ZRM is very common, but these findings are not associated with imaging abnormalities, representing an apparent low risk of injury to the UUT. There was also no association with a history of febrile UTI. The complex neurological damage of these children can determine a myriad of abnormalities in the central, peripheral and autonomic nervous systems that still need to be better elucidated. There is an apparent change in the urodynamic and clinical patterns, determining a reflex urination and low occurrence of UTI after 2 years of age, which may be related to changes in regulation and/or neurovesical and sphincter mechanisms. In conclusion, urodynamic abnormalities in children aged 3 to 5 years with ZRM do not seem to characterize an NB with risks for the UUT. The voiding pattern appears to be reflex, with satisfactory bladder emptying, with a low risk for the UUT. Although these children should keep under urological surveillance, a less invasive approach could be initially offered, reserving UDS for specific cases.

## Figures and Tables

**Table 1:** General clinical and urodynamic findings of 40 children who underwent UDS at OCUH-UPE from October 2018 to March 2020 as part of the MERG-PC.

	n	%
Abnormal urodynamics (n = 40)	40	100.0
Low bladder capacity (n = 40)	37	92.5
Low detrusor compliance (n = 40)	10	25.0
Presence of DO ^1^ (n = 40)	31	77.5
Highest pressure in DO ≥40 cmH_2_O (n = 31)	31	100.0
Presence of urinary leakage in DO (n = 31)	30	96.8
High detrusor leak-point pressure (n = 39)	11	28.2
High postvoid residual volume (n = 40)	8	20.0
Abnormal ultrasonography (n = 40)	3	7.5
Abnormal urethrocystography (n = 5)	0	0.0
Abnormal scintigraphy (n = 5)	0	0.0
Previous febrile UTI ^2^ (n = 40)	10	25.0
Intestinal constipation (n = 40)	27	67.5
Motor deficit (n = 21)	18	85.7
Abnormal appendicular tone (n = 34)	33	97.1
Abnormal axial tone (n = 34)	30	88.2
Convulsions (n = 33)	25	75.8
Distal arthrogryposis (n = 37)	24	64.9
Generalized arthrogryposis (n = 37)	19	51.4
Abnormal EEG ^3^ (n = 15)	14	93.3
Abnormal electroneuromyography (n = 5)	0	0.0

^1^ DO = detrusor overactivity; ^2^ UTI = urinary tract infection; ^3^ EEG = electroencephalography.

**Table 2:** Urodynamic parameters of 40 children undergoing UDS at OCUH-UPE from October 2018 to March 2020 as part of the MERG-PC.

Participant	Age (Years)	Sex	Bladder Capacity (mL)	Detrusor Compliance (mL/cmH_2_O)	Detrusor Overactivity (DO)	Highest Pressure in DO (cmH_2_O)	Urinary Leakage in DO	Detrusor Leak-point Pressure (cmH_2_O)	Postvoid Residual Volume (mL)
1	3	M	120	20	+	60	+	50	30
2	3	M	35	35	+	150	+	35	15
3	3	M	60	12	+	150	+	50	0
4	4	F	50	25	+	100	+	25	0
5	4	M	25	25	+	125	+	10	0
6	4	M	50	25	+	90	+	45	15
7	4	F	25	25	-	NA ^1^	NA	1	10
8	4	M	30	30	+	125	+	25	9
9	4	F	50	1	-	NA	NA	50	20
10	4	M	110	28	+	55	+	30	0
11	4	M	25	25	+	63	+	10	15
12	4	F	110	37	-	NA	NA	15	0
13	4	M	33	33	+	100	+	59	10
14	4	M	17	2	+	75	+	25	4
15	4	M	125	13	+	75	+	55	80
16	4	F	25	25	+	75	+	71	0
17	4	M	33	33	+	137	+	30	0
18	4	F	180	30	+	75	-	NA	180
19	4	M	30	15	+	95	+	50	0
20	4	F	25	25	+	80	+	42	0
21	4	F	23	23	-	NA	NA	1	10
22	4	F	21	21	+	138	+	38	0
23	4	M	130	4	-	NA	NA	30	10
24	4	F	22	22	+	50	+	36	0
25	4	M	25	25	+	100	+	70	13
26	4	M	27	27	+	100	+	38	10
27	4	F	30	30	-	NA	NA	1	0
28	4	M	13	13	+	125	+	10	7
29	4	M	90	45	+	63	+	25	0
30	4	M	75	3	+	100	+	50	8
31	4	F	35	35	+	100	+	30	0
32	4	F	22	22	+	138	+	35	0
33	4	M	95	48	+	63	+	38	0
34	4	F	50	50	+	50	+	25	5
35	4	M	20	20	+	125	+	25	0
36	4	F	90	3	-	NA	NA	36	10
37	4	F	50	50	+	98	+	25	0
38	5	F	50	2	-	NA	NA	21	0
39	5	M	75	38	+	73	+	35	10
40	5	M	264	23	-	NA	NA	8	50
			Mean ± SD ^2^: 59.1 ± 51.4	Mean ± SD: 24.2 ± 13.0		Mean ± SD: 95.3 ± 30.3		Mean ± SD: 32.2 ± 17.7	Mean ± SD: 13.0 ± 31.0

^1^ NA = not applicable; ^2^ SD = standard deviation.

**Table 3:** Association between urodynamic findings and prior febrile UTI ^1^ in 40 children who underwent UDS at the OCUH-UPE from October 2018 to March 2020 as part of the MERG-PC.

Urodynamic Findings	Prior Febrile UTI		*p*
	Present n (%)	Absent n (%)	
**Bladder capacity (n = 40)**			0.012
≥65% da EBC ^2^	3 (100.0%)	0 (0.0%)	
<65% da EBC	7 (18.9%)	30 (81.1%)	
**Detrusor compliance (n = 40)**			0.401
≥20 mL/cmH_2_O	9 (30.0%)	21 (70.0%)	
<20 mL/cmH_2_O	1 (10.0%)	9 (90.0%)	
**DO ^3^ (n = 40)**			1.000
**Present**	8 (25.8%)	23 (74.2%)	
Absent	2 (22.2%)	7 (77.8%)	
**Highest pressure in DO (n = 31)**			
≥40 cmH_2_O	8 (25.8%)	23 (74.2%)	
**Urinary leakage in DO (n = 31)**			0.258
Present	7 (23.3%)	23 (76.7%)	
Absent	1 (100.0%)	0 (0.0%)	
**Detrusor leak-point pressure (n = 39)**			0.693
<40 cmH_2_O	6 (21.4%)	22 (78.6%)	
≥40 cmH_2_O	3 (27.2%)	8 (72.8%)	
**Postvoid residual volume (n = 40)**			0.089
<10% EBC	6 (18.8%)	26 (81.2%)	
≥10% EBC	4 (50.0%)	4 (50.0%)	

^1^ UTI = urinary tract infection; ^2^ EBC = estimated bladder capacity; ^3^ DO = detrusor overactivity.

**Table 4:** Association between urodynamic findings and intestinal constipation in 40 children who underwent UDS at the OCUH-UPE from October 2018 to March 2020 as part of the MERG-PC.

Urodynamic Findings	Intestinal Constipation		*p*
	Present n (%)	Absent n (%)	
**Bladder capacity (n = 40)**			0.538
≥65% da EBC ^1^	3 (100.0%)	0 (0.0%)	
<65% da EBC	24 (64.9%)	13 (31.1%)	
**Detrusor compliance (n = 40)**			0.451
≥20 mL/cmH_2_O	19 (63.3%)	11 (36.7%)	
<20 mL/cmH_2_O	8 (80.0%)	2 (20.0%)	
**DO ^2^ (n = 40)**			1.000
Present	21 (67.8%)	10 (32.2%)	
Absent	6 (66.7%)	3 (33.3%)	
**Highest pressure in DO (n = 31)**			
≥40 cmH_2_O	21 (67.8%)	10 (32.2%)	
**Urinary leakage in DO (n = 31)**			1.000
Present	20 (66.7%)	10 (33.3%)	
Absent	1 (100.0%)	0 (0.0%)	
**Detrusor leak-point pressure (n = 39)**			0.063
<40 cmH_2_O	16 (57.1%)	12 (42.9%)	
≥40 cmH_2_O	10 (91.0%)	1 (9.0%)	
**Postvoid residual volume (n = 40)**			1.000
<10% EBC	22 (68.8%)	10 (31.2%)	
≥10% EBC	5 (62.5%)	3 (37.5%)	

^1^ EBC = estimated bladder capacity; ^2^ DO = detrusor overactivity.

## Data Availability

Data cannot be shared publicly because public availability wouldcompromise patient privacy. De-identified data can be made available upon reasonable request fromqualified investigators by contacting the Programa de Pós-Graduação em Ciências da Saúde (PPGCS) da Universidade de Pernambuco (UPE) at ppg.cienciasdasaude@upe.br.

## Abbreviation

Bladder Capacity = BC
Congenital Zika Syndrome = CZS
Cumulative Incidence = CI
Detrusor Compliance = DC
Detrusor Leak-Point Pressure = DLPP
Detrusor Overactivity = DO
Dimercaptosuccinic Acid = DMSA
Electroencephalogram = EEG
Estimated Bladder Capacity = EBC
Lower Urinary Tract = LUT
Microcephaly Epidemic Research Group = MERG
Neurogenic bladder = NB
Postvoid Residual Volume = PVRV
University of Pernambuco = UPE
Upper Urinary Tract = UUT
Urinary Tract Infection = UTI
Urodynamic Study = UDS
Zika-Related Microcephaly = ZRM
Zika Virus = ZIKV
